# Supplementary material for: Multi-Omics Analysis of Glioblastoma Cells’ Sensitivity to Oncolytic Viruses
Source: Cancers (Basel). 2021 Oct 20;13(21):5268. doi: 10.3390/cancers13215268 (PMC8582528; doi:10.3390/cancers13215268)
Supplement: Supplementary file 1 [file cancers-13-05268-s001.zip › SI_Figures_S1-S10_Tables_S2&S6.pdf]

Cancers MDPI (www.mdpi.com/journal/cancers)

Supplemental Information

Multi-Omics analysis of glioblastoma cells' sensitivity to oncolytic viruses

Anastasia V Lipatova[1,2], Alesya V Soboleva[1], Vladimir A Gorshkov[3], Julia A Bubis[4], Elizaveta M Solovyeva[4], George S Krasnov[1], Dmitry V Kochetkov[1,2], Pavel O Vorobyev[1], Irina Y Ilina[5], Sergei A Moshkovskii[5,6], Frank Kjeldsen[3], Mikhail V Gorshkov[4], Peter M Chumakov**[1,2], Irina A Tarasova*[4]

1 V. A. Engelhardt Institute of Molecular Biology, Russian Academy of Sciences, 119991 Moscow, Russia
2 Center for Precision Genome Editing and Genetic Technologies for Biomedicine, Engelhardt Institute of Molecular Biology, Russian Academy of Sciences, 119991 Moscow, Russia
3 Department of Biochemistry and Molecular Biology, University of Southern Denmark, 5230 Odense M, Denmark
4 V. L. Talrose Institute for Energy Problems of Chemical Physics, N.N. Semenov Federal Research Center for Chemical Physics, Russian Academy of Sciences, 119334 Moscow, Russia
5 Federal Research and Clinical Center of Physical-Chemical Medicine, 119435 Moscow, Russia
6 Pirogov Russian National Research Medical University, 117997 Moscow, Russia

Correspondence: *bioinformatics and data analysis - iatarasova@yandex.ru, **glioblastoma model and oncolytic viruses - chumakovpm@yahoo.com

Content

Figures.
Figure S1. RT-qPCR and Western-blot validation of the knockdown efficiency for MX1, BST2, IFIT3, PLSCR1, and OAS2 and overexpression of PLSCR1.
Figure S2. Statistical design 1 - Type I IFN response of GBM cells cohorts exhibiting intact and defective IFN signals.
Figure S3. Statistical design 2 - Personalized response to IFNα of GBM cells exhibiting intact and defective IFN signals.
Figure S4. Alterations induced by IFNα treatment in "personalized" proteomes.
Figure S5. A core response to type I IFN treatment across the samples with preserved IFN-induced antiviral protection.
Figure S6. IFNα-induced alterations at the level of transcript expression.
Figure S7. KEGG pathway analysis of quantitative transcriptomics data for GBM cultures responsive and silent to IFNα treatment.
Figure S8. Sensitivity to viruses of normal astrocytes and their derivatives with shRNA-mediated silencing of genes for VSV-I, PV3, CA7, CB5, Echo12, and NDV-H2.
Figure S9. KEGG pathway analysis of the IFIT3- and PLSCR1-silent DBTRG-05MG cells relative to the wild type.
Figure S10. Cross-associations between omics data and titration-based measurements.

Tables.
Table S1 contains results of statistical analyses of quantitative changes in cell sensitivity and virus replication and is provided as separate .xlsx file.
Table S2. Oligonucleotides / primers for RT-PCR.
Table S3 contains results of statistical analyses of quantitative changes in transcript expression and is provided as separate .xlsx file.
Table S4 and **Table S5** contain results of statistical analyses of quantitative changes in protein regulation and GO enrichments, respectively. These tables are provided as separate .xlsx files.
Table S6. The intersection between protein and transcript levels in Figure 3a.
Table S7a. Consistency of protein abundances (log2-transformed normalized NSAF) in biological and technical replicates assessed by calculating Pearson and Spearman correlations.
Table S7b. The results of gene enrichment analysis (Gene Ontology, KEGG, Reactome, transcription factors binding data) for genes, for which expression level change (before–after IFN treatment) and sensitivity to VSV-I (before IFN treatment) and VSV replication rates (before IFN treatment) are correlated. Created with g-Profiler tool (https://biit.cs.ut.ee/gprofiler/gost) . Table is provided as separate .xlsx file.

Table S2. Oligonucleotides / primers used in the study.

#	Primer name	Sequence	Purpose
1	RPN1-for	CAC CCT CAA CAG TGG CAA GAA G	RT-PCR
2	RPN1-rev	TGC ATT TCG CTC ACT CTG TCG	RT-PCR
3	GUSB-for	GAT GGA AGA AGT GGT GCG TAG G	RT-PCR
4	GUSB-rev	TTA GAG TTG CTC ACA AAG GTC ACA G	RT-PCR
5	MX1-for	GGC TGT TTA CCA GAC TCC GAC A	RT-PCR
6	MX1-rev	CAC AAA GCC TGG CAG CTC TCT A	RT-PCR
7	BST2-for	TCT CCT GCA ACA AGA GCT GAC C	RT-PCR
8	BST2-rev	TCT CTG CAT CCA GGG AAG CCA T	RT-PCR
9	OAS2-for	GCT TCC GAC AAT CAA CAG CCA AG	RT-PCR
10	OAS2-rev	CTT GAC GAT TTT GTG CCG CTC G	RT-PCR
11	PLSCR1-for	CCT CAG TAT CCA CCG ACA GCA T	RT-PCR
12	PLSCR1-rev	ACA CTG GCT GAT TTG GGA CAG G	RT-PCR
13	IFIT3-for	CCT GGA ATG CTT ACG GCA AGC T	RT-PCR
14	IFIT3-rev	GAG CAT CTG AGA GTC TGC CCA A	RT-PCR
15	MX1-sh dir	5'-phospho-gatccgGCTTTGTGAATTACAGGACATCACGTGATGTCCTGTAATTCACAAAGCtttttg	MX1 knockdown
16	MX1-sh rev	5'-phospho-aattcaaaaaGCTTTGTGAATTACAGGACATCACGTGATGTCCTGTAATTCACAAAGCcg	MX1 knockdown
17	OAS2-sh dir	5'-phospho-gatccgGCAGGGCTCATTGATCTGTATCACGTGATACAGATCAATGAGCCCTGCtttttg	OAS2 knockdown
18	OAS2-sh rev	5'-phospho-aattcaaaaaGCAGGGCTCATTGATCTGTATCACGTGATACAGATCAATGAGCCCTGCcg	OAS2 knockdown

19	PLSCR1-sh dir	5’-phospho-gatccgCAGTTCCCTTTAGACCTTGATCACGTGATCAAGGTCTAAAGGGAACTGtttttg	PLSCR1 knockdown
20	PLSCR1-sh rev	5’-phospho-aattcaaaaaCAGTTCCCTTTAGACCTTGATCACGTGATCAAGGTCTAAAGGGAACTGcg	PLSCR1 knockdown
21	BST2-sh dir	5’-phospho-gatccgCCCAGGAAGCTGGCACATCTTCACGTGAAGATGTGCCAGCTTCCTGGGtttttg	BST2 knockdown
22	BST2-sh rev	5’-phospho-aattcaaaaaCCCAGGAAGCTGGCACATCTTCACGTGAAGATGTGCCAGCTTCCTGGGcg	BST2 knockdown
23	IFIT3-sh dir	5’-phospho-gatccgGCGAAGTCCTTTGAACTCCTACACGTGTAGGAGTTCAAAGGACTTCGCtttttg	IFIT3 knockdown
24	IFIT3-sh rev	5’-phospho-aattcaaaaaGCGAAGTCCTTTGAACTCCTACACGTGTAGGAGTTCAAAGGACTTCGCcg	IFIT3 knockdown
25	GFP2-sh for	5’-phospho-gatccgCGGCGACGTGCACGGCCACAACACGTGTTGTGGCCGTGCACGTCGCCGtttttg	Control lentiviral construct
26	GFP2-sh rev	5’-phospho-aattcaaaaaCGGCGACGTGCACGGCCACAACACGTGTTGTGGCCGTGCACGTCGCCGcg	Control lentiviral construct

a

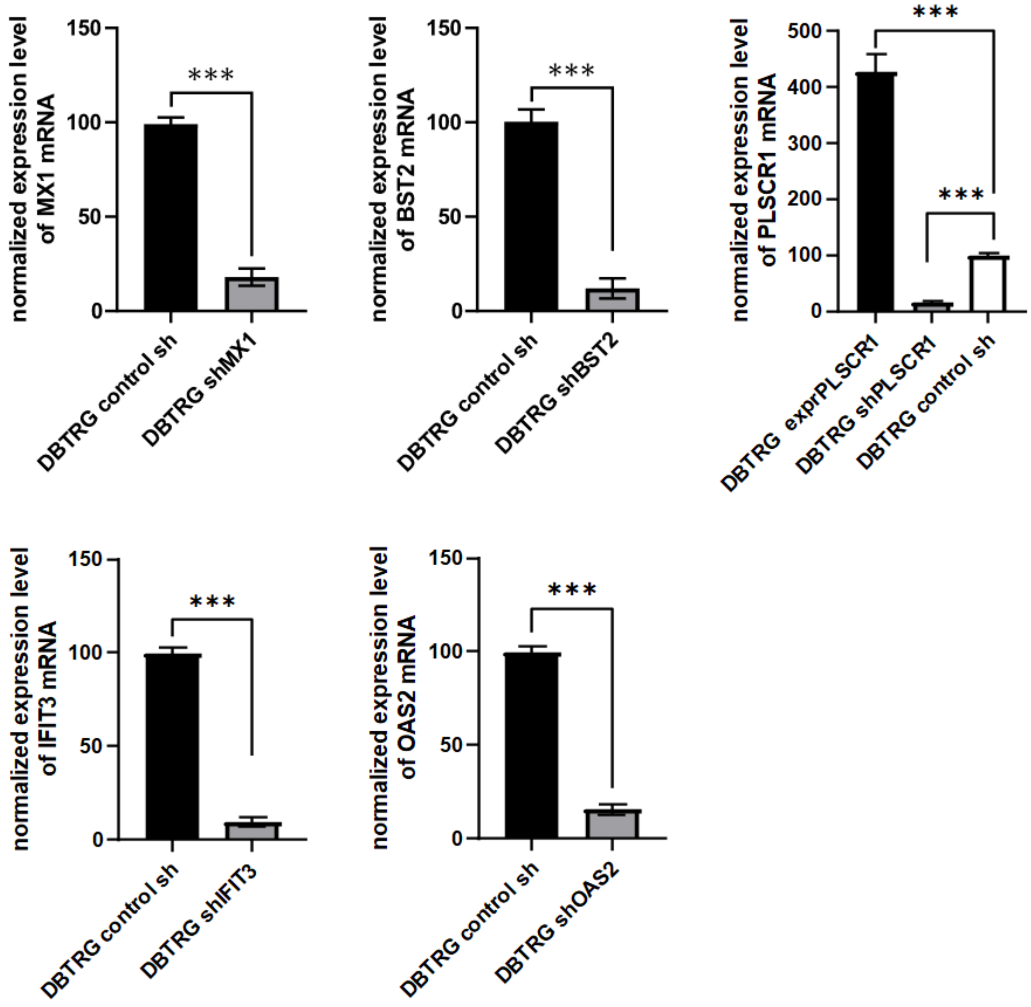
normalized expression level of MX1 mRNA
150
100
50
0
***
DBTRG control sh
DBTRG shMX1
normalized expression level of BST2 mRNA
150
100
50
0
***
DBTRG control sh
DBTRG shBST2
normalized expression level of PLSCR1 mRNA
500
400
300
200
100
0
***
***
DBTRG exprPLSCR1
DBTRG shPLSCR1
DBTRG control sh
normalized expression level of IFIT3 mRNA
150
100
50
0
***
DBTRG control sh
DBTRG shIFIT3
normalized expression level of OAS2 mRNA
150
100
50
0
***
DBTRG control sh
DBTRG shOAS2

b

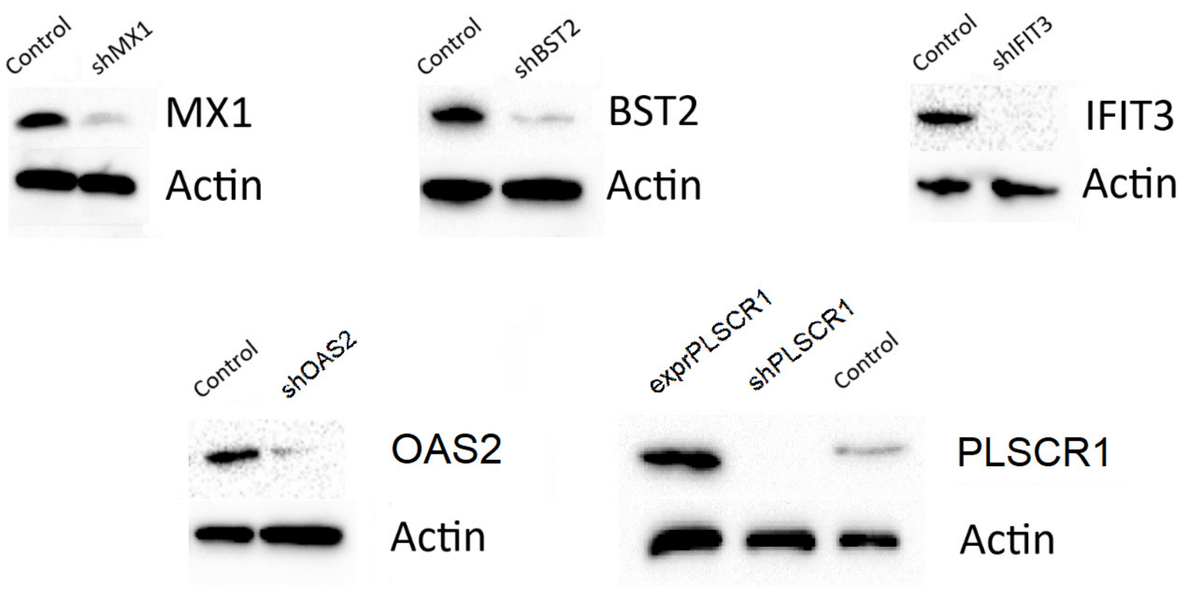

Figure S1. RT-qPCR (**a**) and Western-blot (**b**) validation for shRNA-mediated knockdowns of MX1, BST2, IFIT3, PLSCR1, and OAS2 and overexpression of PLSCR1 in DBTRG-05MG cells. Labels: ***P<0.001 (t-test). Control corresponds to DBTRG-05MG with lentiviral construction expressing non-sense shRNA for GFP2 protein.

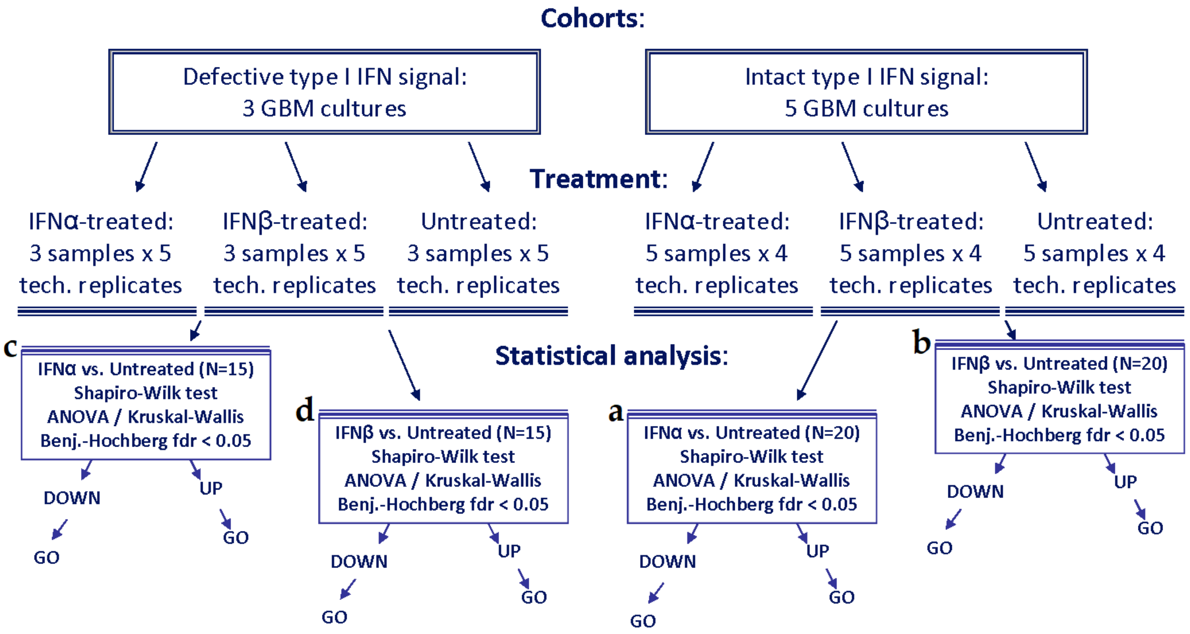

Figure S2. Statistical design 1 - Type I IFN response of GBM cells cohorts exhibiting intact and defective IFN signals. Cohorts were composed as given in **Figure 1a**. Gene ontology (GO) analyses were performed separately for upregulated (UP) and downregulated (DOWN) proteins. Results of GO analyses are provided in **Table S5**. Labels: **a,b** correspond to data presented in **Figure 2a,b**; **c,d** correspond to data shown in **Figure 2c,d**.

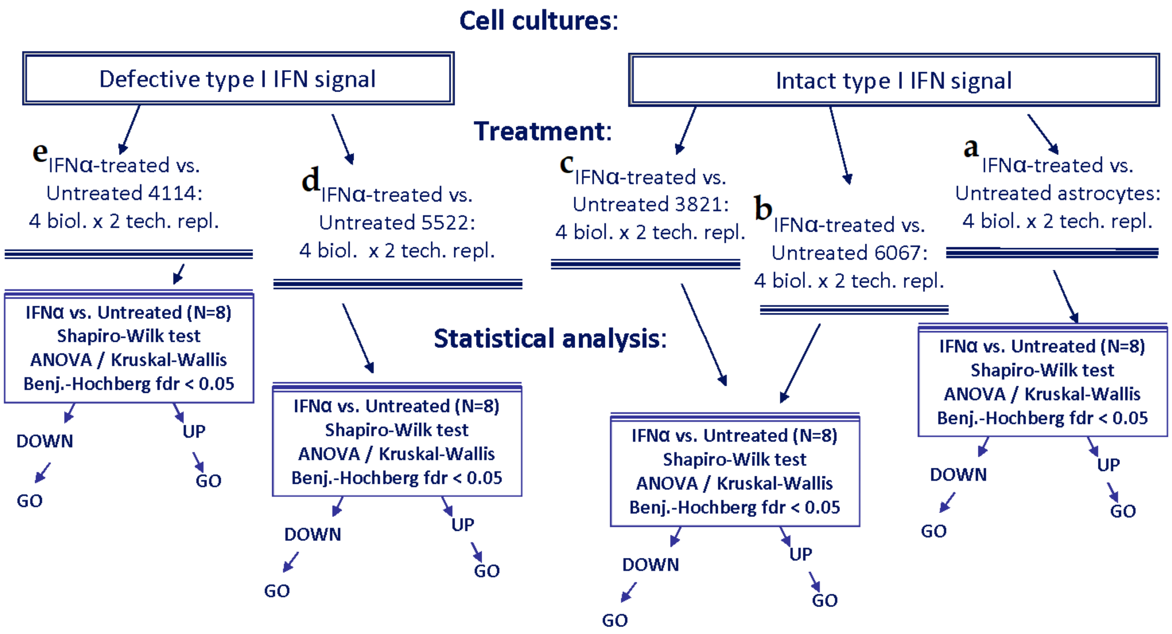

Figure S3. Statistical design 2 - Personalized responses to IFNα of GBM cells exhibiting intact and defective IFN signals and normal astrocytes. Gene ontology (GO) analyses were performed separately for upregulated (UP) and downregulated (DOWN) proteins. Results of GO analyses are provided in **Table S5**. Labels: **a,b,c,d,e** correspond to data presented in **Figure S4a,b,c,d,e**, respectively.

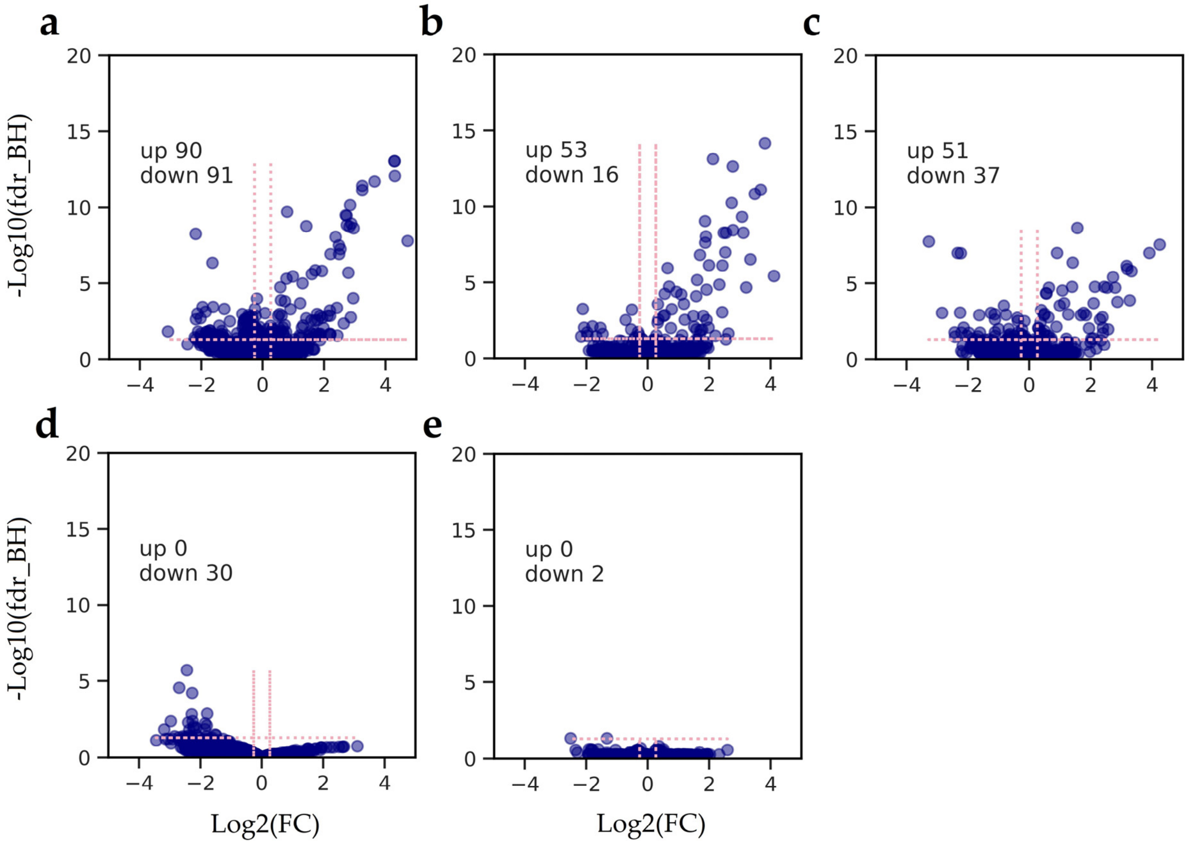

Figure S4. Alterations induced by IFNα treatment in "personalized" proteomes: (**a**) normal astrocytes; (**b**) GBM6067 cells; (**c**) GBM3821 cells; (**d**) GBM5522 cells; (**e**) GBM4114 cells. Dashed lines correspond to statistical (fdr_BH < 0.05) and fold change ($|\log2FC| \geq 0.263$) thresholds used for the determination of differentially regulated proteins. Statistical results are provided in **Table S4**. Imputed NSAF scaling = 1.0.

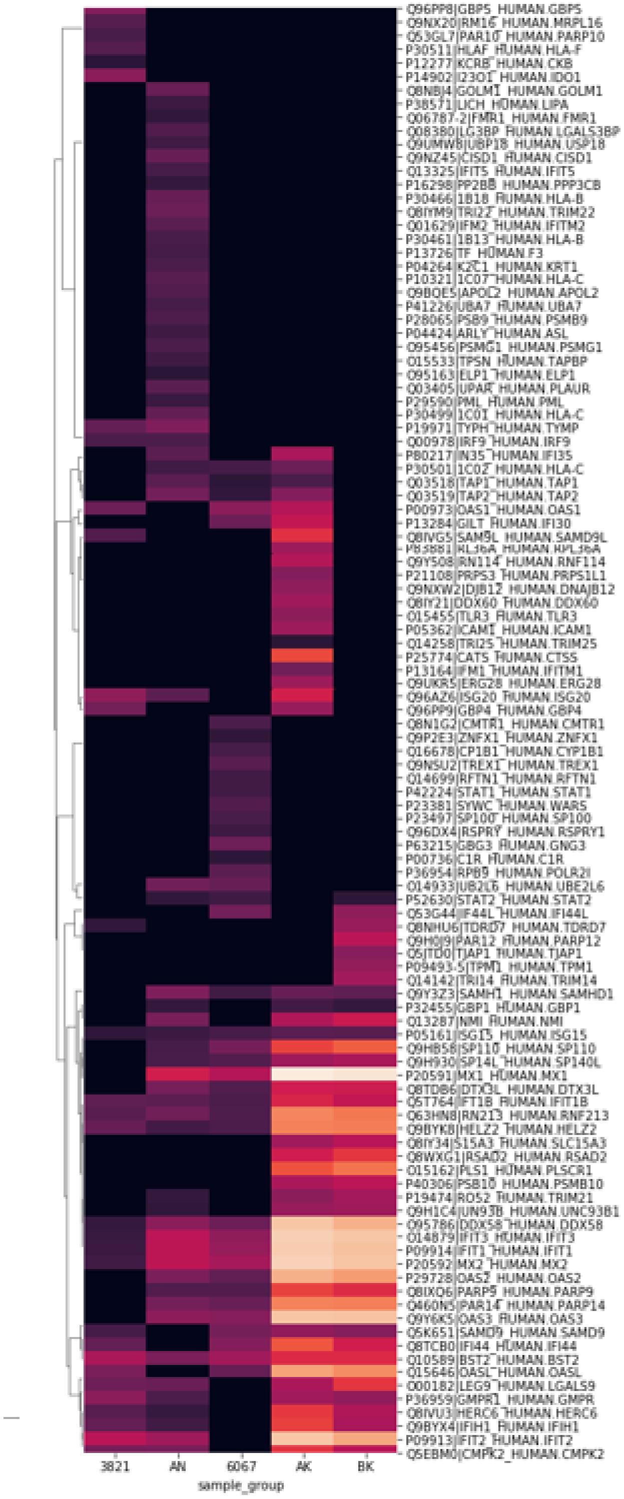

Figure S5. A core response to type I IFN treatment across the samples with preserved IFN-induced antiviral protection. Labels: (AK) response to IFNα in the "Preserved" cohort from **Figure 1a**; (BK) response to IFNβ in "Preserved" cohort from **Figure 1a**; (3821) individual response to IFNα, GBM3821 culture; (6067)

individual response to IFNα, GBM6067 culture; (AN) individual response to IFNα, normal astrocytes. Protein selection satisfies fdr_BH < 0.05 and FC ≥ 2.0.

Table S6. The intersection between protein and transcript levels in Figure 3a.

Gene	log2FC_rna	FDR_rna	log2(FC)_prot	FDR_prot
MX1	9.88854	0.0	9.215449573513146	2.461854266706127e-21
MX2	7.71729	0.0	8.568412506128553	4.0950858611670926e-20
IFIT3	8.32462	0.0	8.520909431267413	3.6220670974760284e-20
IFIT1	9.50028	0.0	8.455145293608009	3.4143281220768996e-20
IFIT2	10.6027	0.0	8.347268241351433	5.4010076363170876e-37
DDX58	6.95411	0.0	8.292109517859334	4.322197947471922e-36
OAS3	6.17899	0.0	8.260882125622318	5.814014242943736e-44
OAS2	6.5604	0.0	7.810840914983264	1.3381332424548157e-36
OASL	9.36349	0.0	7.495120016379276	6.6538195054904815e-28
RNF213	3.94263	0.0	6.908545398105939	4.3095773872410334e-08
PARP14	5.13819	0.0	6.831425151533861	9.294339357090447e-10
HELZ2	5.66453	0.0	6.772066114278189	4.153841452862189e-08
IFI44	5.64144	0.0	5.939844083358767	5.496414221385176e-08
PLSCR1	4.11412	0.0	5.873122939213674	3.850671484832955e-08
CTSS	2.47966	0.0	5.6970673969128205	1.0468066666179733e-07
SP110	4.07248	0.0	5.6115027941483415	2.1005031406155465e-06
PARP9	4.01826	0.0	5.598850526302567	4.933961249687956e-07
IFIH1	7.69481	0.0	5.508037634815996	3.3569655719628126e-06
HERC6	6.41497	0.0	5.396302253094364	2.3616334833272457e-06
CMPK2	8.97698	0.0	5.295953754160941	1.7306266980950006e-05
SAMD9L	5.73309	0.0	5.227359608225009	0.00013010893915393166
BST2	4.77349	0.0	5.045532476584997	1.1194983150192464e-05
IFIT1B	7.27822	0.0	4.935971943540616	2.403106813574076e-06
ISG20	5.20065	0.0	4.782230673913394	0.000235362833437544
RSAD2	11.3544	0.0	4.712094842627391	0.003954438561141535
DTX3L	3.68273	0.0	4.689171401382402	4.612007756708089e-05
OAS1	7.92537	0.0	4.07471961513737	0.02210284954298042
RNF114	1.75389	2.01367E-41	4.058078366421877	0.0019423305745560027
NMI	3.38011	0.0	4.003775539061478	0.006240795783700184
LGALS9	4.57283	0.0	3.894352685051832	0.012143131020476922
PSMB10	2.24859	2.28218E-40	3.889921284089764	0.01215216988230213
IFI35	4.10312	0.0	3.836555233307207	0.033892664348644616
GMPR	4.79908	0.0	3.6965862063337855	0.013261398522239416
SLC15A3	3.66876	0.0	3.6756429680524416	0.004755297067710619
DDX60	3.85044	0.0	3.6305893343873215	0.012143131020476922
SP140L	2.4484	0.0	3.6031098416359986	0.008441373718553033
ICAM1	2.90142	0.0	3.568641491936553	0.017043380262832124
UNC93B1	1.54798	2.30779E-19	3.513914849354536	0.0031875181956160037
GBP4	9.20392	0.0	3.4619762969568817	0.03298791976016185
SAMD9	4.80019	0.0	3.363723955597232	0.001643026396239627
TLR3	5.62741	0.0	3.2425551837399285	0.03156329307093264

TRIM21	3.57279	0.0	3.2292295781139777	0.0384172689927205
TAP2	3.29872	0.0	3.0857947959824767	0.016375300340519743
IFITM1	3.05096	0.0	2.637809181328927	0.013652843197151713
HLA-C	1.20371	2.60356E-19	2.5350998157999878	0.033710932029595964
SAMHD1	4.04093	0.0	2.4279365231787984	0.0002513816564275716
ISG15	6.83242	0.0	2.1725952321346633	0.00027212192656423246
TAP1	4.51065	0.0	1.8057753380551764	0.017043380262832124
GBP1	5.84744	0.0	1.5960733314665756	9.502333673836054e-08
TRIM25	2.22031	0.0	1.017830711648527	2.066080319568266e-05
PNPT1	3.79186	0.0	0.9997231474244441	0.0069018771236951494
PML	2.81492	0.0	0.9088757593495992	2.400329835317142e-07
EIF2AK2	2.99012	0.0	0.9027240669116668	2.974674207612645e-09
STAT2	2.47324	0.0	0.897223770771953	0.005752582963402845
WARS	4.21652	0.0	0.8549097609988995	1.3569732271999761e-05
APOL2	4.80897	1.18753E-40	0.7763583513510864	0.00027212192656423246
HLA-H	1.35116	9.04047E-19	0.7039190070873041	1.6280024035169678e-05
STAT1	2.68534	0.0	0.6756813932450825	0.00015956534304545518
LGALS3BP	1.35953	2.63369E-19	0.6067913662210935	0.0014993961167550382
TAPBP	1.15133	2.47741E-17	0.588213780803842	0.031109641051011036
CNP	2.56139	0.0	0.5875506704940134	0.005691852421139973
LAP3	3.34996	0.0	0.5824673290301234	0.00021134998851069967
HLA-C	1.20371	2.60356E-19	0.5751625873706079	0.0059043715123928715
ADAR	1.98929	0.0	0.5544170951233898	0.04593463355564716
B2M	0.580054	4.79929E-5	0.4025316495473053	0.018795245799940904
NAPA	1.09457	3.85848E-17	0.2755107083892455	0.002254721170352373
TNFRSF10D	-1.99718	1.76218E-28	-3.1500694289005526	0.010794908701486713

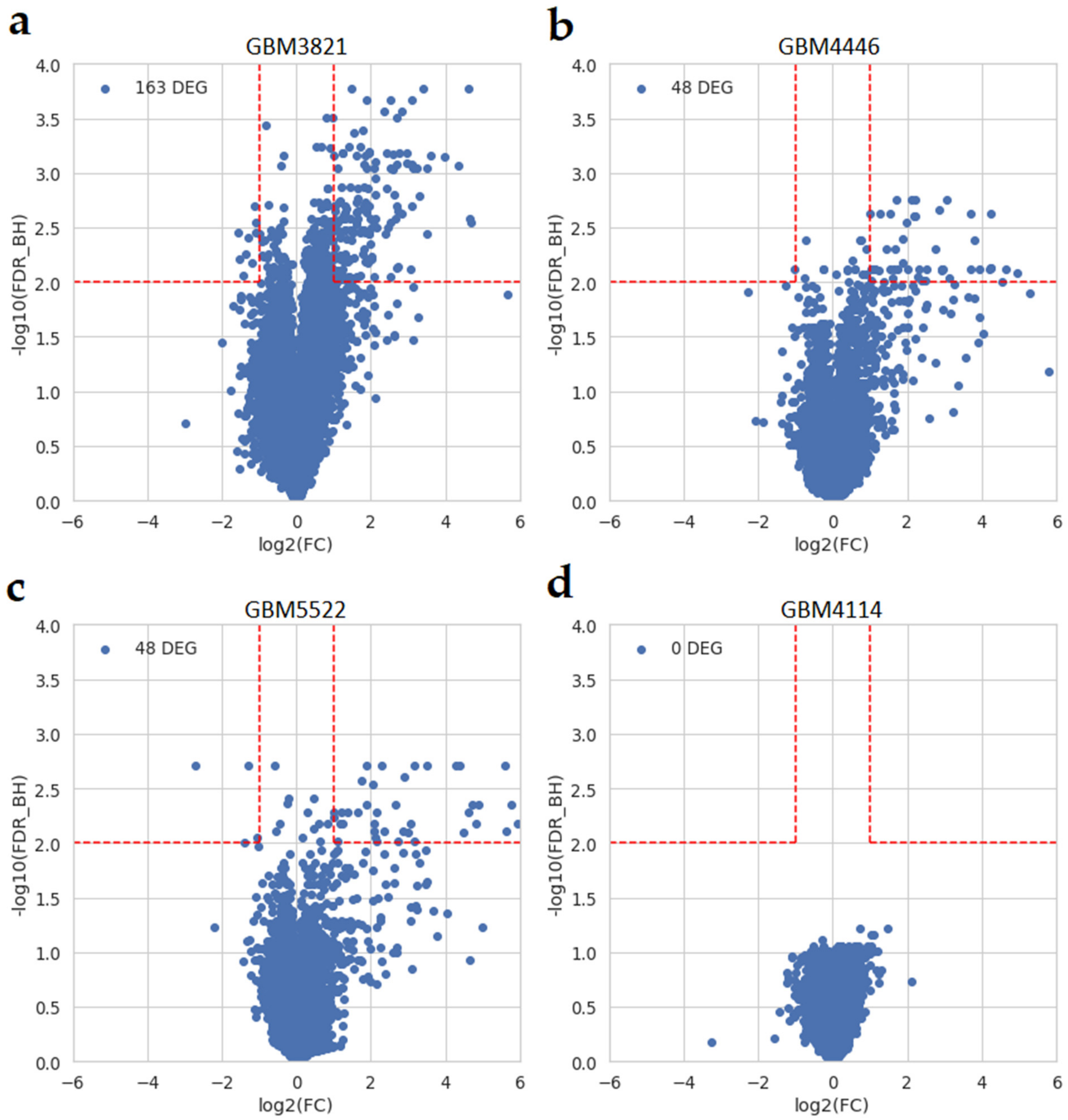

Figure S6. Alterations induced by IFNα treatment in transcriptomes of GBM3821, GBM4446, GBM5522, and GBM4114 cells. Dashed lines correspond to statistical thresholds (fdr_BH < 0.01 and |FC|>2) used for the determination of differentially expressed genes (DEG).

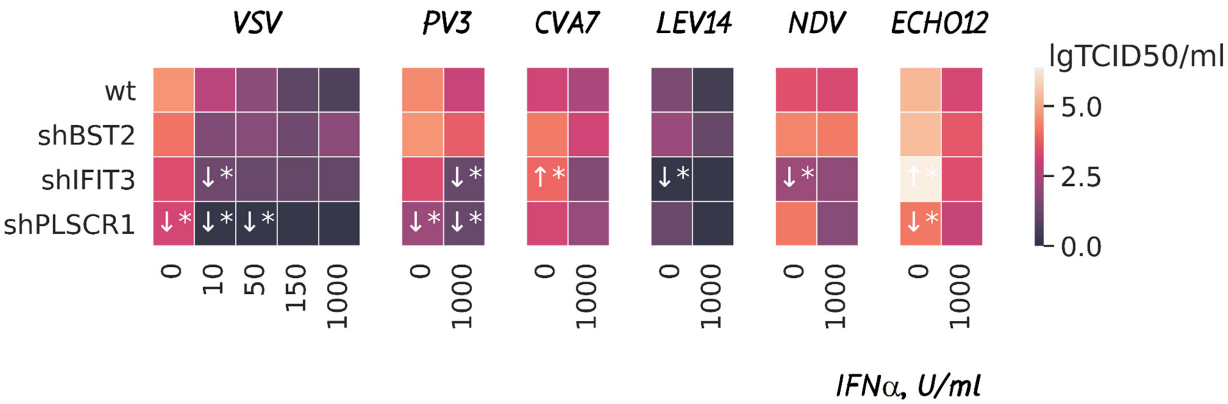

Figure S7. Sensitivity to viruses of normal astrocytes and their derivatives with shRNA-mediated silencing of genes for VSV-I, PV3, CA7, CB5, Echo12, and NDV-H2. The sensitivity was measured in quadruplicates for a range of IFN*α* concentrations by virus titration in the sensitive RD cells. Cell cultures were treated with IFN*α* for 24 h, followed by infection with a virus. The tissue culture infectious dose required to reach the fifty percent endpoint, lgTCID50/ml, was measured 24 h after infection. Labels: ↑*↓* denotes a significant increase or decrease in cell sensitivity with a *t*-test p-value < 0.05.

a

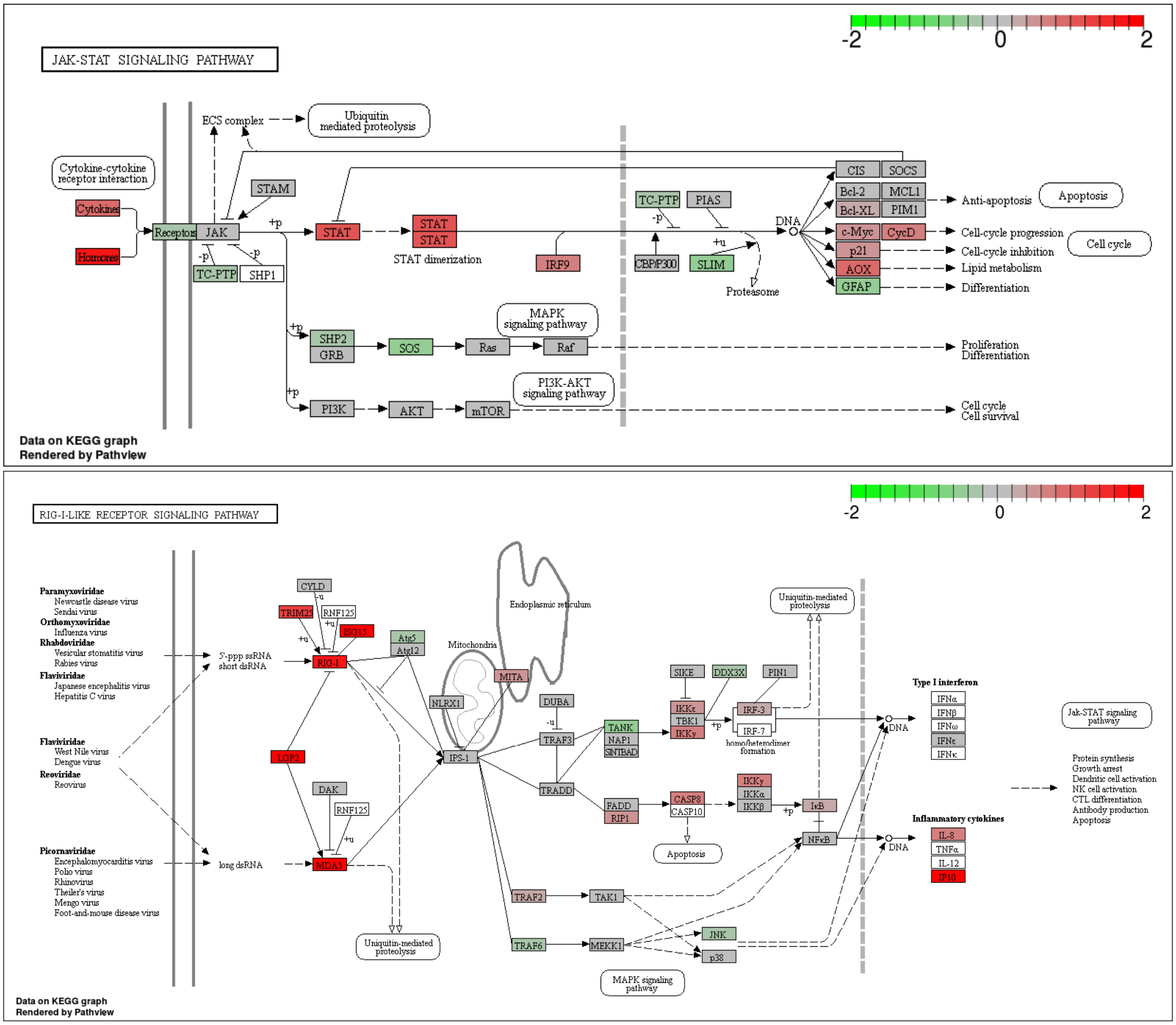

JAK-STAT SIGNALING PATHWAY
-2
0
2
ECS complex
Ubiquitin mediated proteolysis
Cytokine-cytokine receptor interaction
Cytokines
Hormones
Receptors
JAK
STAM
TC-PTP
SHP1
STAT
STAT
STAT
STAT dimerization
IRF9
TC-PTP
PIAS
CBP/P300
SLIM
Proteasome
DNA
CIS
SOCS
Bcl-2
MCL1
Bcl-XL
PIM1
c-Myc
CycD
p21
AOX
GFAP
Anti-apoptosis
Apoptosis
Cell-cycle progression
Cell cycle
Cell-cycle inhibition
Lipid metabolism
Differentiation
SHP2
GRB
SOS
Ras
Raf
MAPK signaling pathway
Proliferation
Differentiation
PI3K
AKT
mTOR
PI3K-AKT signaling pathway
Cell cycle
Cell survival
Data on KEGG graph
Rendered by Pathview
RIG-I-LIKE RECEPTOR SIGNALING PATHWAY
-2
0
2
Paramyxoviridae
Newcastle disease virus
Sendai virus
Orthomyxoviridae
Influenza virus
Rhabdoviridae
Vesicular stomatitis virus
Rabies virus
Flaviviridae
Japanese encephalitis virus
Hepatitis C virus
Flaviviridae
West Nile virus
Dengue virus
Reoviridae
Reovirus
Picornaviridae
Encephalomyocarditis virus
Polio virus
Rhinovirus
Theiler's virus
Mengo virus
Foot-and-mouth disease virus
5'-ppp ssRNA
short dsRNA
long dsRNA
CYLD
TRIM25
RNF125
ISG15
RIG-I
LGP2
DAK
RNF125
MDA5
Atg5
Atg12
Mitochondria
Endoplasmic reticulum
NLRX1
MITA
IPS-1
DUBA
TRAF3
TRADD
TANK
NAP1
SINTBAD
SIKE
DDX3X
PIN1
IKKε
TBK1
IKKγ
IRF-3
IRF-7
homo/heterodimer formation
Uniquitin-mediated proteolysis
FADD
RIP1
CASP8
CASP10
Apoptosis
IKKγ
IKKα
IKKβ
IκB
NFκB
DNA
DNA
Type I interferon
IFNα
IFNβ
IFNω
IFNε
IFNκ
Jak-STAT signaling pathway
Protein synthesis
Growth arrest
Dendritic cell activation
NK cell activation
CTL differentiation
Antibody production
Apoptosis
Inflammatory cytokines
IL-8
TNFα
IL-12
IP10
TRAF2
TAK1
TRAF6
MEKK1
JNK
p38
MAPK signaling pathway
Uniquitin-mediated proteolysis
Data on KEGG graph
Rendered by Pathview

b

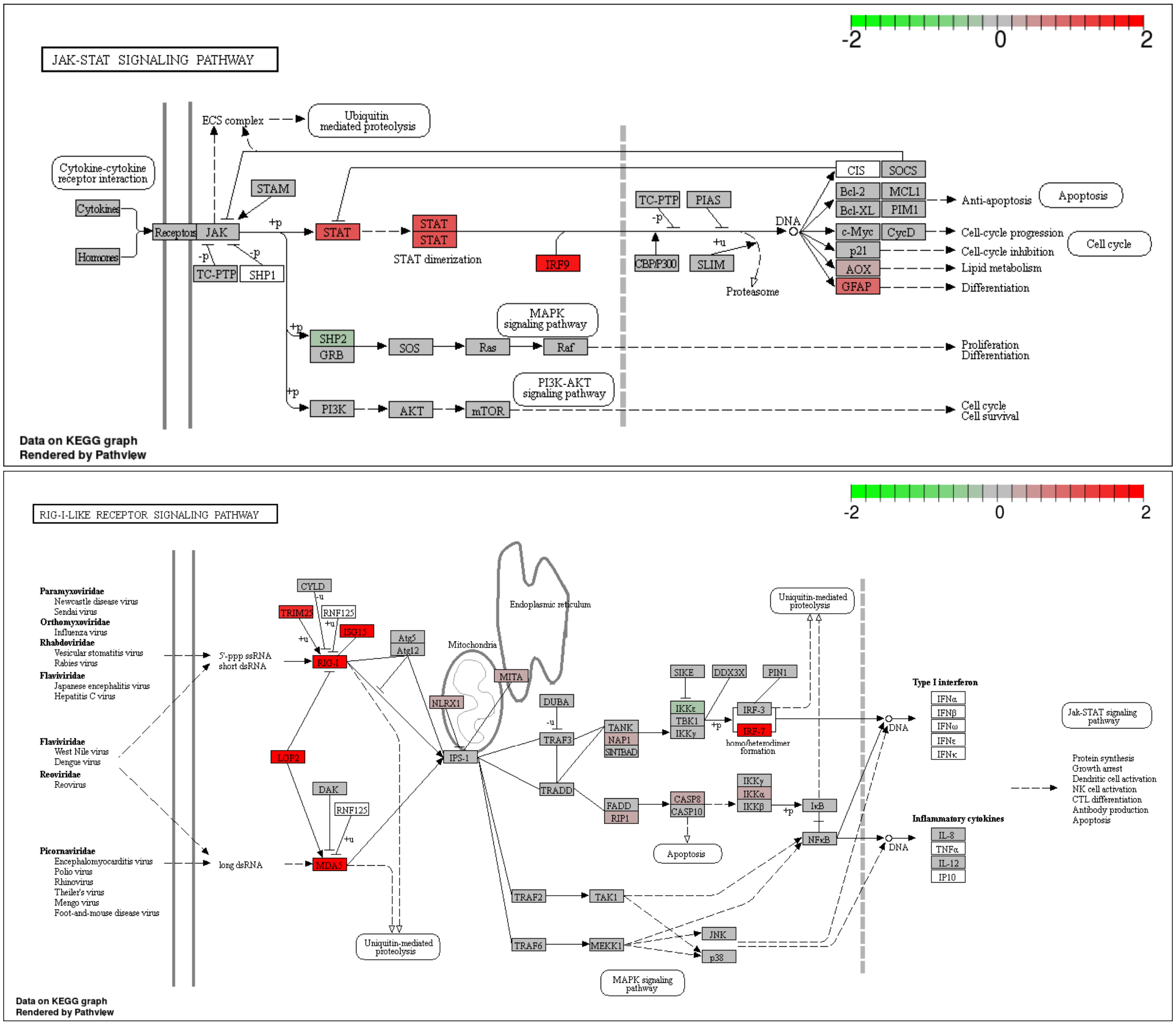

-2
0
2
JAK-STAT SIGNALING PATHWAY
ECS complex
Ubiquitin mediated proteolysis
Cytokine-cytokine receptor interaction
Cytokines
Hormones
Receptors
JAK
STAM
+p
-p
TC-PTP
SHP1
STAT
STAT
STAT
STAT dimerization
IRF9
CIS
SOCS
Bcl-2
MCL1
Bcl-XL
PIM1
c-Myc
CycD
p21
AOX
GFAP
Anti-apoptosis
Apoptosis
Cell-cycle progression
Cell cycle
Cell-cycle inhibition
Lipid metabolism
Differentiation
TC-PTP
PIAS
CBP/P300
SLIM
+u
DNA
Proteasome
MAPK signaling pathway
SHP2
GRB
SOS
Ras
Raf
Proliferation
Differentiation
PI3K-AKT signaling pathway
PI3K
AKT
mTOR
Cell cycle
Cell survival
Data on KEGG graph
Rendered by Pathview
-2
0
2
RIG-I-LIKE RECEPTOR SIGNALING PATHWAY
Paramyxoviridae
Newcastle disease virus
Sendai virus
Orthomyxoviridae
Influenza virus
Rhabdoviridae
Vesicular stomatitis virus
Rabies virus
Flaviviridae
Japanese encephalitis virus
Hepatitis C virus
Flaviviridae
West Nile virus
Dengue virus
Reoviridae
Reovirus
Picornaviridae
Encephalomyocarditis virus
Polio virus
Rhinovirus
Theiler's virus
Mengo virus
Foot-and-mouth disease virus
5'-ppp ssRNA
short dsRNA
long dsRNA
CYLD
TRIM25
RNF125
ISG15
RIG-I
Atg5
Atg12
LGP2
DAK
RNF125
MDA5
Uniquitin-mediated proteolysis
Endoplasmic reticulum
Mitochondria
MITA
NLRX1
IPS-1
DUBA
TRAF3
TRADD
TANK
NAP1
SINTBAD
SIKE
DDX3X
PIN1
IKKε
TBK1
IKKγ
IRF-3
IRF-7
homo/heterodimer formation
Uniquitin-mediated proteolysis
FADD
RIP1
CASP8
CASP10
Apoptosis
IKKγ
IKKα
IKKβ
IκB
NFκB
DNA
Type I interferon
IFNα
IFNβ
IFNω
IFNε
IFNκ
Jak-STAT signaling pathway
Protein synthesis
Growth arrest
Dendritic cell activation
NK cell activation
CTL differentiation
Antibody production
Apoptosis
Inflammatory cytokines
IL-8
TNFα
IL-12
IP10
TRAF2
TAK1
TRAF6
MEKK1
JNK
p38
MAPK signaling pathway
Data on KEGG graph
Rendered by Pathview

c

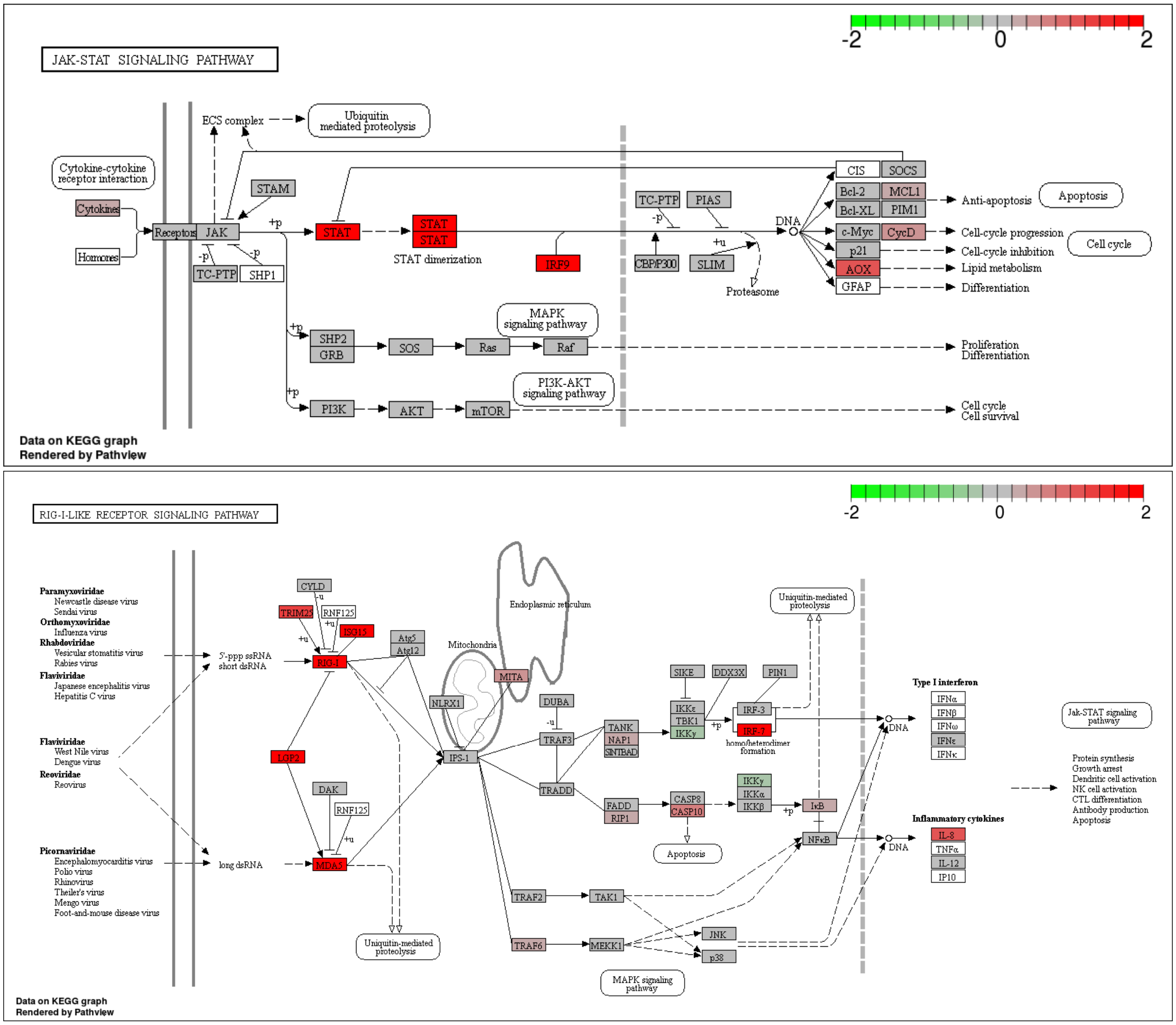

-2
0
2
JAK-STAT SIGNALING PATHWAY
ECS complex
Ubiquitin mediated proteolysis
Cytokine-cytokine receptor interaction
Cytokines
Hormones
Receptors
JAK
STAM
TC-PTP
SHP1
STAT
STAT
STAT
STAT dimerization
IRF9
TC-PTP
PIAS
CBP/P300
SLIM
Proteasome
DNA
CIS
SOCS
Bcl-2
MCL1
Bcl-XL
PIM1
c-Myc
CycD
p21
AOX
GFAP
Anti-apoptosis
Apoptosis
Cell-cycle progression
Cell cycle
Cell-cycle inhibition
Lipid metabolism
Differentiation
MAPK signaling pathway
SHP2
GRB
SOS
Ras
Raf
Proliferation
Differentiation
PI3K-AKT signaling pathway
PI3K
AKT
mTOR
Cell cycle
Cell survival
Data on KEGG graph
Rendered by Pathview
-2
0
2
RIG-I-LIKE RECEPTOR SIGNALING PATHWAY
Paramyxoviridae
Newcastle disease virus
Sendai virus
Orthomyxoviridae
Influenza virus
Rhabdoviridae
Vesicular stomatitis virus
Rabies virus
Flaviviridae
Japanese encephalitis virus
Hepatitis C virus
Flaviviridae
West Nile virus
Dengue virus
Reoviridae
Reovirus
Picornaviridae
Encephalomyocarditis virus
Polio virus
Rhinovirus
Theiler's virus
Mengo virus
Foot-and-mouth disease virus
5'-ppp ssRNA
short dsRNA
long dsRNA
CYLD
TRIM25
RNF125
ISG15
RIG-I
Atg5
Atg12
LGP2
DAK
RNF125
MDA5
Uniquitin-mediated proteolysis
Endoplasmic reticulum
Mitochondria
MITA
NLRX1
IPS-1
DUBA
TRAF3
TANK
NAP1
SINTBAD
TRADD
FADD
RIP1
CASP8
CASP10
Apoptosis
SIKE
DDX3X
PIN1
IKKε
TBK1
IKKγ
IRF-3
IRF-7
homo/heterodimer formation
Uniquitin-mediated proteolysis
IKKγ
IKKα
IKKβ
IκB
NFκB
DNA
DNA
Type I interferon
IFNα
IFNβ
IFNω
IFNε
IFNκ
Jak-STAT signaling pathway
Protein synthesis
Growth arrest
Dendritic cell activation
NK cell activation
CTL differentiation
Antibody production
Apoptosis
Inflammatory cytokines
IL-8
TNFα
IL-12
IP10
TRAF2
TAK1
TRAF6
MEKK1
JNK
p38
MAPK signaling pathway
Data on KEGG graph
Rendered by Pathview

d

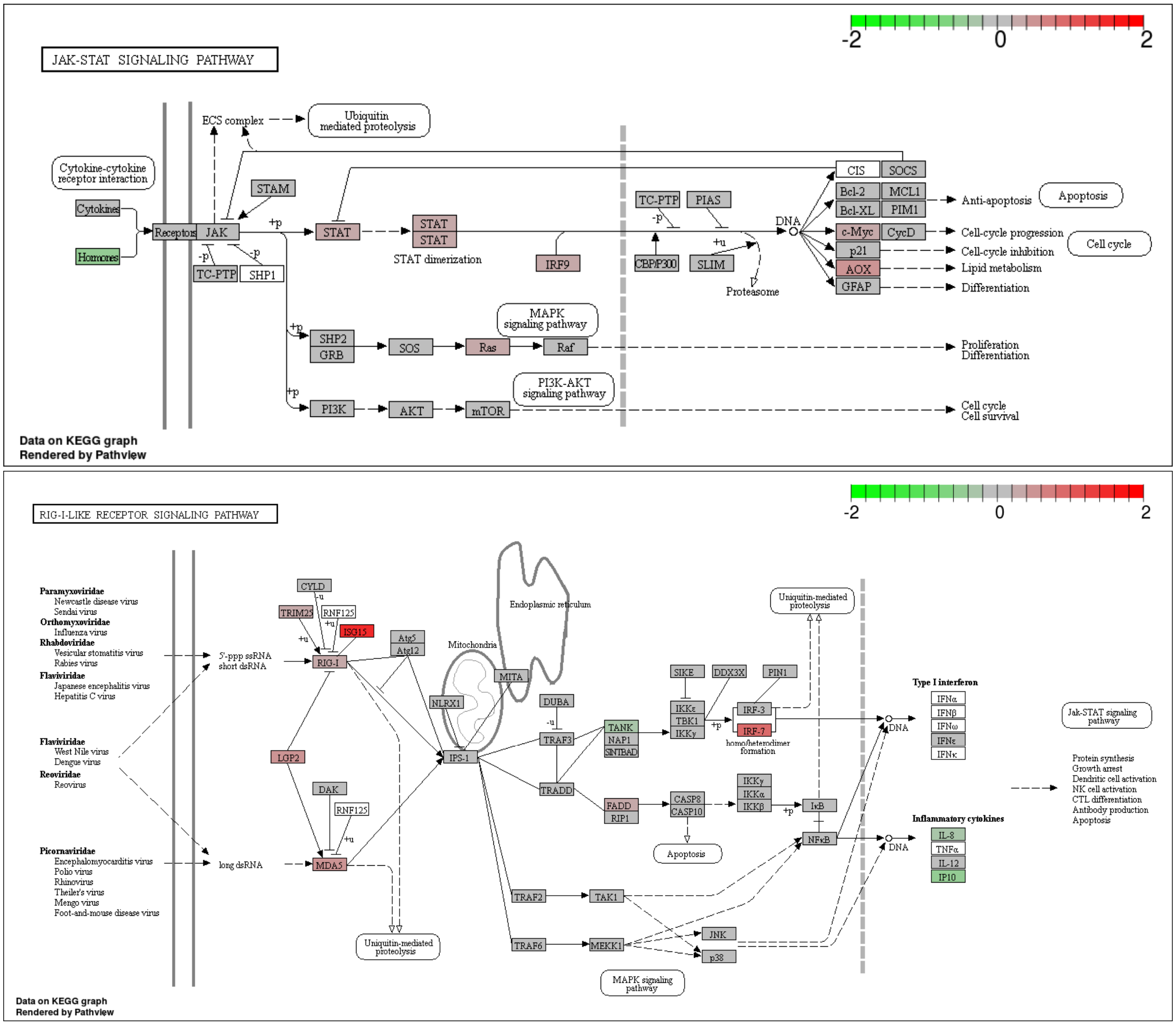

Figure S8. KEGG pathway analysis of quantitative transcriptomics data for GBM cultures responsive and silent to IFNα treatment: (**a**) responsive GBM3821 cells, (**b**) responsive GBM4446 cells, (**c**) responsive GBM5522 cells, and (**d**) silent GBM4114 cells.

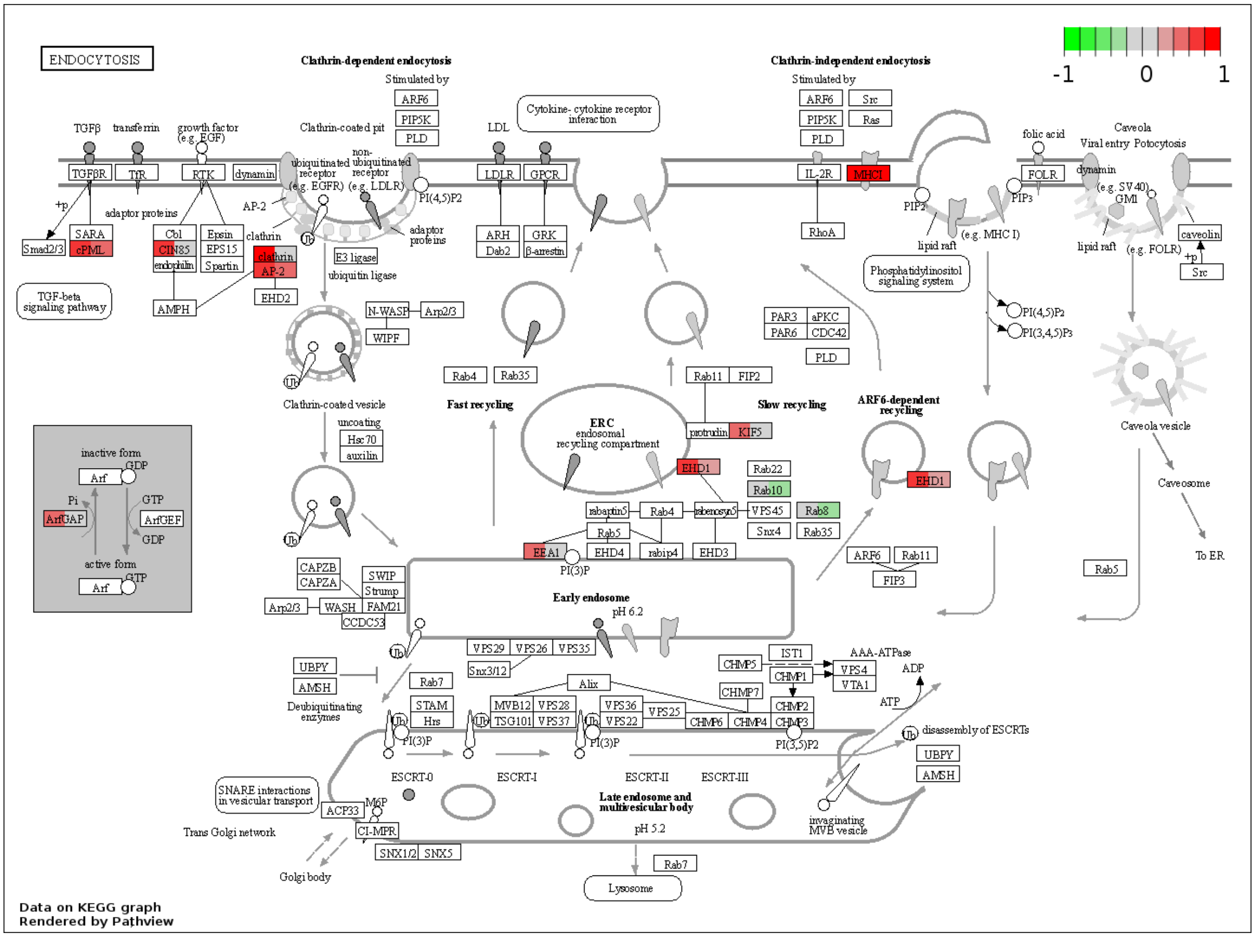

ENDOCYTOSIS
Clathrin-dependent endocytosis
Clathrin-independent endocytosis
Stimulated by
ARF6
PIP5K
PLD
Src
Ras
Cytokine- cytokine receptor interaction
Caveola
Viral entry Potocytosis
TGFβ
transferrin
growth factor (e.g. EGF)
Clathrin-coated pit
LDL
folic acid
TGFβR
TfR
RTK
dynamin
LDLR
GPCR
IL-2R
MHCI
FOLR
SARA
cPML
Smad2/3
Cbl
CIN85
endophilin
Epsin
EPS15
Spartin
clathrin
AP-2
EHD2
E3 ligase
ubiquitin ligase
ARH
Dab2
GRK
β-arrestin
RhoA
Phosphatidylinositol signaling system
TGF-beta signaling pathway
AMPH
N-WASP
Arp2/3
WIPF
Clathrin-coated vesicle
uncoating
Hsc70
auxilin
Rab4
Rab35
Fast recycling
Rab11
FIP2
Slow recycling
ERC
endosomal recycling compartment
protrudin
KIF5
EHD1
Rab22
Rab10
Rab8
Rab35
PAR3
PAR6
aPKC
CDC42
PLD
ARF6-dependent recycling
Caveola vesicle
Caveosome
To ER
Rab5
PI(4,5)P2
PI(3,4,5)P3
inactive form
active form
Arf
GDP
GTP
Pi
ArfGAP
ArfGEF
rabaptin5
Rab4
Rab5
rabenosyn5
VPS45
Snx4
EEA1
EHD4
rabip4
EHD3
ARF6
Rab11
FIP3
PI(3)P
Early endosome
pH 6.2
CAPZB
CAPZA
SWIP
Strump
Arp2/3
WASH
FAM21
CCDC53
VPS29
VPS26
VPS35
Snx3/12
UBPY
AMSH
Deubiquitinating enzymes
Rab7
STAM
Hrs
MVB12
VPS28
TSG101
VPS37
Alix
VPS36
VPS22
VPS25
CHMP6
CHMP4
CHMP2
CHMP3
CHMP5
CHMP1
CHMP7
IST1
VPS4
VTA1
AAA-ATPase
ADP
ATP
disassembly of ESCRTs
PI(3,5)P2
ESCRT-0
ESCRT-I
ESCRT-II
ESCRT-III
Late endosome and multivesicular body
pH 5.2
SNARE interactions in vesicular transport
ACP33
M6P
CI-MPR
SNX1/2
SNX5
Trans Golgi network
Golgi body
invaginating MVB vesicle
Lysosome
-1
0
1
Data on KEGG graph
Rendered by Pathview

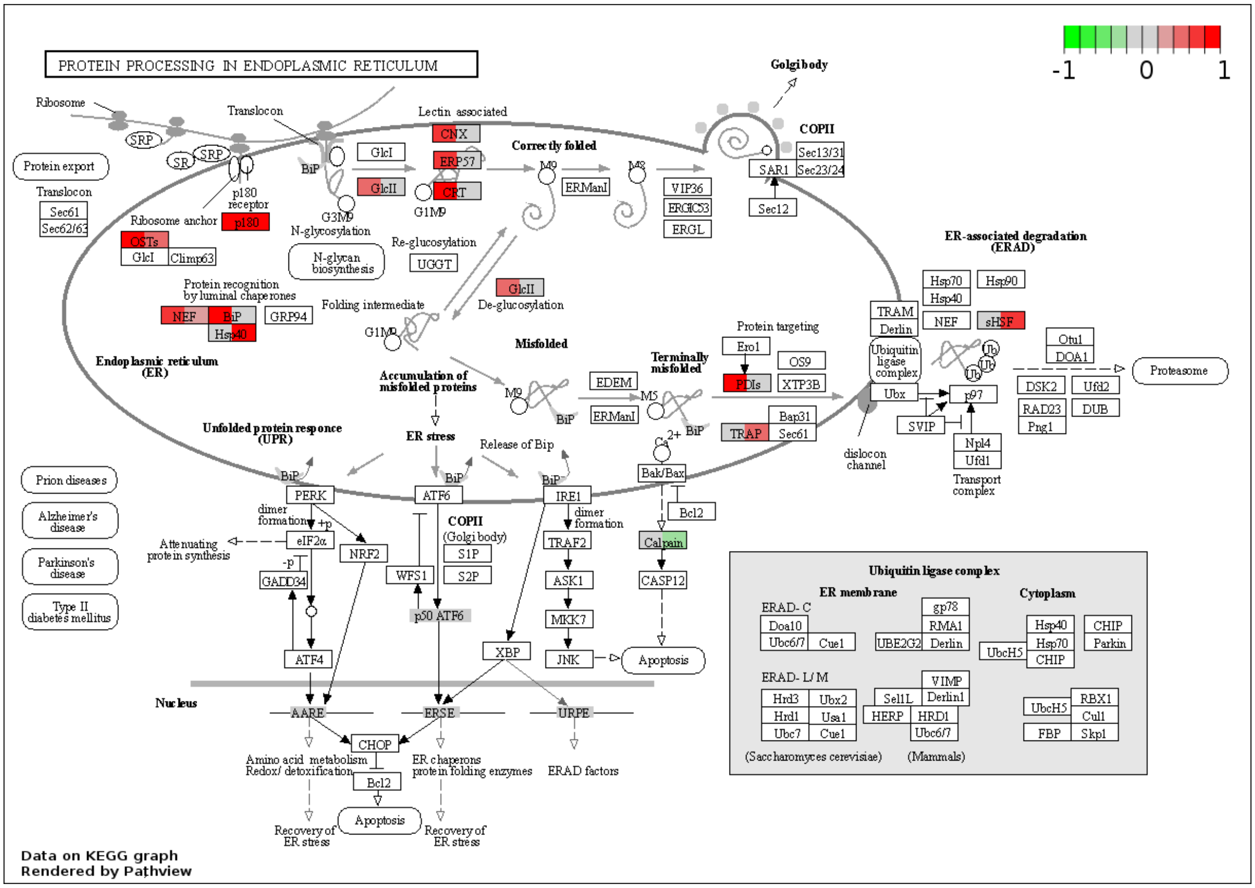

Figure S9. KEGG pathway analysis of the IFIT3- and PLSCR1-silent DBTRG-05MG cells relative to the wild type.

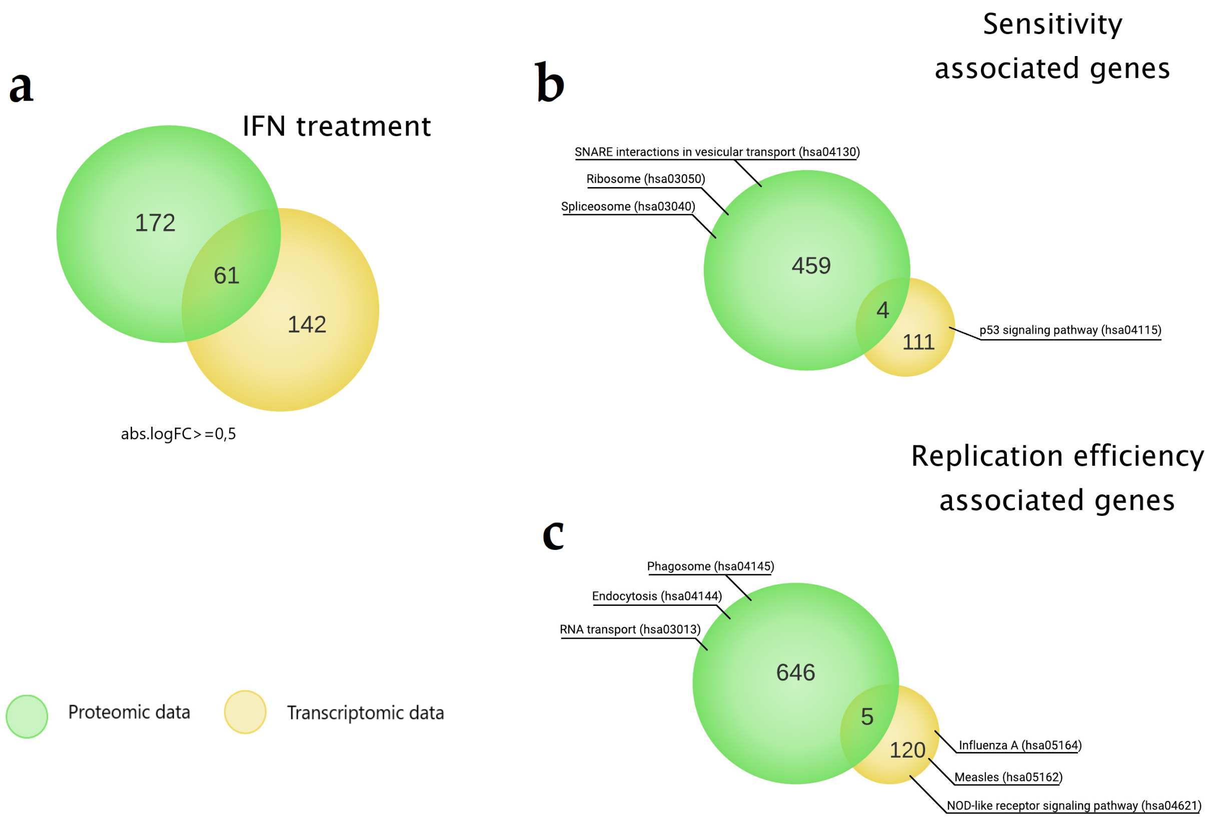

Figure S10. Cross-associations between omics data and titration-based measurements: (**a**) differentially expressed features induced by IFN treatment (primary GBM cells, Welch *t*-test with correction for multiple comparisons, p-value < 0.05); (**b**) genes, for which expression level change (before–after IFN treatment) is correlated with differences in cell sensitivity to VSV as high as abs(R) ≥ 0.75, are involved in biological processes associated with cell sensitivity; (**c**) genes, for which expression level change (before–after IFN treatment) is correlated with differences in VSV replication as high as abs(R) ≥ 0.75, are involved in biological processes associated with virus replication.
